# Evaluation of Brassica Vegetables as Potential Feed for Ruminants

**DOI:** 10.3390/ani9090588

**Published:** 2019-08-21

**Authors:** Trinidad de Evan, Andrea Vintimilla, Carlos N. Marcos, María José Ranilla, María Dolores Carro

**Affiliations:** 1 Departamento de Producción Agraria, Escuela Técnica Superior de Ingeniería Agronómica, Agroalimentaria y de Biosistemas, Universidad Politécnica de Madrid, Ciudad Universitaria, 28040 Madrid, Spain; 2 Departamento de Producción Animal, Universidad de León. 24071 León, Spain; 3 IGM (CSIC-ULE), Finca Marzanas s/n. 24346 Grulleros, León, Spain

**Keywords:** Brassica vegetables, in vitro rumen fermentation, methane: in situ rumen degradability, intestinal digestibility

## Abstract

**Simple Summary:**

According to Food and Agriculture Organization of the United Nations (FAO) estimations, nearly 50% of the initial production of vegetables and fruits is lost or wasted. Losses can happen at different stages of the food chain supply, but mainly at agricultural production (cultivation and harvest), distribution, and consumption. The wasted vegetables represent a significant volume that could be used for ruminant feeding, but before using them in practice, it is necessary to evaluate their nutritive value. In this study, we analyzed the chemical composition, in vitro rumen fermentation, and intestinal digestibility of wastes of four types of cabbages: Brussels sprouts, white cabbage, Savoy cabbage, and red cabbage. All cabbages wastes had high moisture content, but their dry matter was rich in protein and sugars and was rapidly and extensively fermented in the rumen. The in vitro results of this study provided useful information for including cabbage wastes in ruminant diets, indicating that Brussel sprouts could be included up to 24% of the concentrate of a dairy sheep diet, replacing cereals and soybean meal, without negatively affecting rumen fermentation. In vivo studies are required to confirm these results.

**Abstract:**

The objective of this study was to analyze the chemical composition, in vitro ruminal fermentation, and intestinal digestibility of discarded samples of four Brassica vegetables: Brussels sprouts (BS), white cabbage, Savoy cabbage, and red cabbage, and to assess the effects of including increasing amounts of BS in the concentrate of a dairy sheep diet on in vitro fermentation, CH_4_ production, and in situ degradation of the diets. All cabbages had low dry matter content (DM; <16.5%), but their DM had high crude protein (19.5–24.8%) and sugars (27.2–41.4%) content and low neutral detergent fiber (17.5–28%) and was rapidly and extensively fermented in the rumen. Rumen degradability of protein at 12 h of in situ incubation was greater than 91.5% for all cabbages, and in vitro intestinal digestibility of protein ranged from 61.4 to 90.2%. Replacing barley, corn, and soybean meal by 24% of dried BS in the concentrate of a diet for dairy sheep (40:60 alfalfa hay:concentrate) increased in vitro diet fermentation and in situ degradability of DM and protein, and reduced in vitro CH_4_/total volatile fatty acid ratio. In vivo trials are necessary to confirm these results.

## 1. Introduction

The production of fresh vegetables and fruits for human consumption is continuously growing worldwide, but it is estimated that about 46% of the initial production is lost or wasted at different stages of the food chain supply in the European Union, increasing this figure up to more than 55% in Africa and Latin America [1]. These losses can happen at different stages of the food chain supply, such as agricultural production (cultivation and harvest), postharvest handling and storage, processing, distribution, and consumption. In the industrialized regions of the world, losses at agricultural production dominate and are mostly due to postharvest vegetable and fruit grading caused by quality standards set by retailers, but wastes at distribution and consumption are also substantial [1]. These wastes represent a significant volume that could be used for animal feeding, thus generating economic benefits for the farmers due to reduced feeding costs and contributing to alleviate the environmental problems associated with their elimination [2,3]. These wastes have a high moisture content, being highly fermentable and rapidly perishable, which is the most important limitation for its further utilization in animal feeding. However, they can be sun-dried before getting introduced in ruminant diets [4] or ensiled [5].

Discarded Brassica vegetables are frequently used in practice as part of ruminant diets in temperate agricultural systems [6] and developing countries [4], although information on their nutritive value is limited. Some previous studies [7,8,9,10,11] have analyzed the chemical composition of different Brassica vegetables, but in most of them, only a single sample of a Brassica vegetable was assessed. On the other hand, information on digestive utilization of Brassica vegetables is very scarce, although some in vitro experiments [11,12,13] have shown that cabbage leaves can be extensively fermented in the rumen.

Brassica vegetables contain multiple secondary compounds [14], and some of them can be toxic to animals and reduce their growth and productive performance when fed in high amounts [15], but it has been shown that ruminal microorganisms can detoxify these compounds when animals are introduced gradually to cabbage feeding [6]. Some of these compounds might modulate ruminal fermentation, but to our best knowledge, their possible effect on ruminal CH_4_ production has not yet been investigated. Therefore, the objective of this study was to analyze the chemical composition, in vitro ruminal fermentation, and intestinal digestibility of discarded samples of four Brassica vegetables: Brussels sprouts (BS; *Brassica oleracea var. gemmifera*), white cabbage (WC; *Brassica oleracea var. Capitata* f. alba), Savoy cabbage (SC; *Brassica oleracea var.* capitata f. sabauda), and red cabbage (RC; *Brassica oleracea var. capitata f. rubra*). Besides, the effects of including increasing amounts of BS in the concentrate of a dairy sheep diet on in vitro fermentation and CH_4_ production, as well as on in situ degradation of the diet, were assessed.

## 2. Materials and Methods

Animals used in this study were cared and managed following the Spanish regulations for experimental animal protection, and experimental procedures were approved by the Institutional Animal Care and Use Committee of the Comunidad Autónoma de Madrid (PROEX 035/17).

### 2.1. Samples of Cabbage Wastes

Three samples of each BS were obtained from two local markets on three different weeks between October and December 2017. Samples obtained the same week were pooled, cut into pieces, and dried at 40 °C until constant weight. All samples were ground to pass a 2 mm sieve before starting the trials.

### 2.2. Animals and Feeding

Four adult rumen-fistulated Lacaune sheep (64.3 ± 2.11 kg body weight) were used as rumen fluid donors for both in vitro incubations and for in situ incubations. Animals were individually housed in floor pens, had free access to freshwater. Sheep were fed a diet composed of grass hay and concentrate in 2:1 ratio, which contained 114 g of crude protein (CP), 365 g of neutral detergent fiber (NDF), and 160 g of acid detergent fiber (ADF) per kg DM. The diet was administered daily in two equal portions at 9:00 and 18:00 at a restricted level (45 g dry matter (DM)/kg body weight ^0.75^).

### 2.3. Experimental Design

The study comprised two different experiments. Chemical composition, in vitro rumen fermentation, and in vitro nitrogen (N) intestinal digestibility (IVND) of the vegetable wastes were determined in Experiment 1. Experiment 2 was conducted to analyze the effects of including increasing amounts of BS in the diet on in vitro fermentation and in situ degradation of the diets. Samples for chemical analyses and in vitro incubations were ground at 1 mm, whereas 2 mm samples were used for in situ incubations.

#### 2.3.1. Experiment 1: In Vitro Ruminal Fermentation and Intestinal Digestibility of Discarded Vegetables

Two different in vitro ruminal incubations were carried out on different days using the Goering and Van Soest procedure [16], as detailed in [17]. The first incubation lasted for 144 h and was conducted to assess the gas production kinetics of the samples, whereas the main fermentative parameters were determined in a 24 h incubation. For both incubations, ruminal contents of each sheep were collected immediately before the morning feeding, strained through four layers of cheesecloth, and independently mixed with pre-warmed (39 °C) culture medium ([16] without trypticase and NH_4_HCO_3_) in 1:4 proportion under CO_2_ flushing. Two hundred milligram of DM of each sample was weighed into 60-mL vials, which were filled up with 20 mL of the mixture of rumen fluid and culture medium using a peristaltic pump (Watson-Marlow 520UIP31; Watson-Marlow Fluid Technology Group, Cornwall, United Kingdom). Vials were sealed with rubber stoppers and incubated at 39 °C. Gas production was measured at 3, 6, 9, 12, 15, 22, 26, 31, 36, 48, 58, 72, 96, 120, and 144 h with a pressure transducer (Delta Ohm DTP704-2BGI, Herter Instruments SL, Barcelona, Spain) and a plastic syringe. Bottles without substrate (blanks; two per inoculum) were included to correct the endogenous gas production values. Besides, one sample of each barley, sugar beet pulp, and wheat distiller’s dried grains with solubles (DDGS) was included in the incubation to serve as a reference, as they are feeds widely used in ruminant feeding. The potential in vitro degradability of DM (PDMD) was determined by weighing in triplicate 300 mg of each sample into Ankom Corp #57 bags (30 µm pore size; Ankom Technology Corp., Fairport, NY, United States) and incubating them in the mixture of ruminal fluid and the culture medium previously described in an Ankom Daisy II incubator at 39 °C under continuous rotation for 144 h. After 144 h, bags were washed with cold water, dried at 60 °C for 48 h, and weighed to calculate PDMD.

The second in vitro incubation lasted for 24 h, and after this period, gas production was measured, vials were uncapped, and their content was homogenized before measuring the pH, and 3 mL of vials content that was mixed with 3 mL of 0.5 M HCl was taken and frozen (−20 °C ) until volatile fatty acid (VFA) and NH_3_-N analyses. In each of the two incubations, there were four replicates per sample by using the ruminal fluid from each sheep independently as inoculum.

The analysis of in vitro N intestinal digestibility was performed, as described by Gargallo et al., [18] using the batch incubator Daisy^II^ (Ankom Technology, Fairport, NY, USA). Two gram of each sample (2 mm ground) was weighed in duplicate into nylon bags (5 x 15 cm; 46 μm pore size), and bags were incubated in the rumen of each sheep immediately before the morning feeding for 12 h. After withdrawal, bags were rinsed to eliminate feed particles and immediately frozen (−20 °C) for one week. Finally, bags were thawed, washed with cold water in a turbine washing machine (3 cycles of 5 min each), and frozen before being lyophilized and weighed. The incubation residues were analyzed for N content to calculate the in situ N degradability after 12 h incubation.

Residues of 12 h incubation were pooled by vegetable sample, and 0.3 g of each was carefully weighed in duplicate into Ankom R510 bags cut in half (50 μm pore size; 5 × 5 cm). Bags were incubated in a buffer of pH 1.9 (0.1 N HCl) and containing 1 g/L of pepsin (P-7000, Sigma, St. Louis, MO, USA) for 1 h in the Daisy^II^ incubator. The bags were taken out, washed under running with tap water, and then further incubated for 24 h in a buffer of pH 7.75 (0.5 M KH_2_PO_4_) containing 3 g/L of pancreatin (P-7545, Sigma, St. Louis, MO, USA) and 50 ppm of thymol. Both incubations were performed at 39 °C. Finally, bags were washed with cold water in a turbine washing machine (2 cycles of 5 min each), dried at 40 °C for 72 h, and weighed. Incubation residues were pooled by sample and sheep and analyzed for N concentration to calculate the in vitro intestinal digestibility of CP.

#### 2.3.2. Experiment 2: In Vitro Fermentation and In Situ Degradability of Diets Containing Increasing Amounts of Brussels Sprouts

The objective of this experiment was to analyze the effects of including increasing amounts of dried BS in the concentrate of a dairy sheep diet on in vitro rumen fermentation and in situ degradation. All diets were composed of 40% of alfalfa hay and 60% concentrate (fresh matter basis). The control concentrate was high in cereals to be representative of those used in dairy ruminants feeding in practice. Three additional concentrates were formulated by replacing different amounts of corn, barley, and soybean meal in control concentrate with 8, 16, or 24 g of dried BS per 100 g of concentrate. The experimental concentrates were formulated to have similar CP and NDF content to the control one, as later described. A composited sample of the three BS samples tested in Experiment 1 was used, and it contained 92, 24.8, 17.8, 10.7, and 2.85 g of organic matter (OM), CP, NDF, ADF, and ether extract (EE) per 100 of DM, respectively.

Using the 24 h in vitro incubation procedure, described in Experiment, 1400 mg of DM were incubated into 120 mL vials, which were filled up with 40 mL of the ruminal fluid and culture medium mixture. The amount of sample was increased to get enough gas for CH_4_ analyses. After 8 h of incubation, gas production was measured, and a sample (10 mL) was taken into evacuated tubes for CH_4_ analysis. The content of each vial was then homogenized, and 1 mL was sampled using a syringe and mixed with 1 mL 0.5 M HCl before being frozen (20 °C) until VFA and NH_3_-N analyses. After 24 h of incubation, samples of gas and vials content were taken, as described before, for analyses of CH_4,_ VFA, and NH_3_-N, and the pH of vials content was measured. Similar to Experiment 1, there were four replicates per diet.

The in situ procedure for measuring degradation kinetics of DM and N was carried out, as described by Belverdy et al. [19]. Briefly, 3 g of each diet (2 mm ground) was weighed into nylon bags (5 × 15 cm; 46 μm pore size) and incubated in the rumen of each sheep immediately before the morning feeding for 2, 4, 8, 16, 24, 48, and 72 h. Incubations were repeated on two different days in each sheep. After withdrawal, bags were rinsed to eliminate feed particles and immediately frozen (−20 °C) for one week. Finally, bags were thawed, washed with cold water in a turbine washing machine (3 cycles of 5 min each), dried at 40 °C for 72 h, and weighed. The same washing procedure was applied to two bags of each diet to obtain the washing loss value (0 h incubation), and the residue remaining into the bags was considered as the insoluble fraction. The incubation residues were pooled for sheep and incubation time and analyzed for N content.

### 2.4. Chemical Analyses

The procedures of the analysis of the chemical composition of vegetable samples, barley, sugar beet pulp, DDGS, and feed ingredients of experimental diets have been detailed by Marcos et al. [16]. Concentrations of NH_3_-N were measured by the colorimetric method, described by Weatherburn [20]. Concentrations of CH_4_ in the gas produced and of VFA in the vial’s content were analyzed by gas chromatography following the procedures described by García-Martínez et al. [21] and Martínez et al. [22], respectively. All chemical analyses were performed in duplicate.

### 2.5. Calculations and Statistical Analyses

Gas production values were fitted to the model: Gas = A (1 − e ^(−c (t − *lag*))^) using the NLIN procedure of Statistical Analysis System (SAS) [23]. In this model, A is the potential gas production, *c* is the fractional rate of gas production, *lag* is the time before the onset of gas production, and t is the measurement time. The average gas production rate (AGPR) was considered the rate of gas production between the start of the incubation and the time at which the 50% of potential gas production was reached and was calculated as AGPR = A *c*/[2 (ln2 + *c lag*)]. Finally, the effective degradability of DM (DMED) was calculated for a rumen digesta passage of 0.042 per h (kp) according to the following equation DMED = [(PDMD × *c*)/(*c* + *K*p)] e ^(^^−^*^c^* ^×^^lag)^. This passage rate corresponds to a rumen retention time of 24 h, which was used for the in vitro incubations, and can be found in sheep fed at moderate intake levels [24]. Finally, the amount of apparently fermented OM (AFOM) was calculated from acetate, propionate, and butyrate production in each vial, as described by Demeyer [25].

Data from Experiment 1 were analyzed using the PROC MIXED of SAS [23] as a mixed model, including the fixed effect of cabbage type and the random effect of the inoculum. Data from Experiment 2 were analyzed as a mixed model, in which the effect of diet was fixed, and the effect of the inoculum was random. The linear and quadratic effects of BS inclusion were assessed by non-orthogonal polynomial contrasts. The level of significance was set at *p* < 0.05, and *p* < 0.10 values were considered as trends. The Tukey’s test was used to compare means when a significant effect of sample or diet was detected. The PROC CORR of SAS [23] was used to analyze the relationships between the chemical composition of vegetables and both gas production and fermentation parameters by linear regression.

## 3. Results and Discussion

### 3.1. Experiment 1: Chemical Composition, In Vitro Ruminal Fermentation, and In Vitro Intestinal Digestibility of Vegetables

The chemical composition of cabbages and the feeds used as a reference is shown in Table 1. Chemical composition of reference feeds was in the range of values previously reported [26]. As expected, all cabbages had low DM content, ranging from 6.60 to 16.3%. Brussels sprouts had greater (*p* < 0.05) DM, OM, and CP content than the other cabbages, but lower (*p* < 0.05) EE, NDF, and ADF content than Savoy and red cabbage. Although differences did not reach significance (*p* > 0.05), BS had numerically lower values of lignin/NDF ratio (<1.5%) and neutral detergent insoluble CP concentration (<5% of total CP) than the other vegetables (>6% and 13.5%, respectively). All vegetables had low concentrations of lignin (<2.5% on DM basis). Other authors [8,9,10] have reported similar concentrations of OM, CP, NDF, ADF, and EE for different cabbages types, but lower values of sugars (about 21% of DM). In contrast, other studies [7,8,9,10,11,12] have reported lower CP content. The differences in chemical composition compared to results reported in the literature might be due to the stage of growth, season, species and variety, soil types, and growth environment. Compared with the feeds used as a reference, Brassica vegetables had EE, NDF, and lignin contents similar to those in barley and DDGS, but greater sugars content.

**Figure 1:**
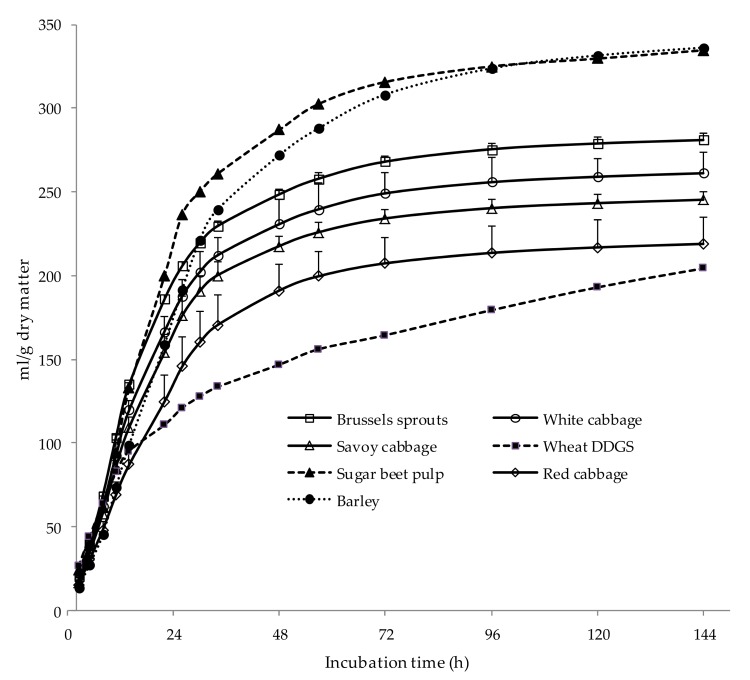
Gas production kinetics (mean values and standard errors) of Brussels sprouts (□), white cabbage (○), Savoy cabbage (∆), red cabbage (◊), barley (●), sugar beet pulp (▲), and wheat DGGS (■). DDGS: dried distiller grains with solubles.

Gas production and fermentation parameters of vegetable samples are shown in Table 2, whereas gas production curves are presented in Figure 1. Brussels sprouts had the greatest (*p* < 0.05) potential gas production (A), fractional rate of gas production (c), average gas production rate (AGPR), and DMED, but there were no differences (*p* = 0.168) among cabbages in the time until gas production started (*Lag*). These results are in agreement with the differences observed in the chemical composition of cabbages. Brussels sprouts had a high content of sugars (41.4% of DM), which are easily and rapidly degraded in the rumen, as well as low content in poorly lignified NDF. There were few differences among white, Savoy, and red gas production parameters, although red cabbage had lower (*p* < 0.05) *c* and AGPR values than Savoy cabbage. Our results are in accordance with others [11,12,13], who observed high potential gas production values (ranging from 167 to 296 mL of gas per g DM) when different types of cabbages were fermented in vitro.

All cabbages had lower potential gas production than barley and sugar beet pulp, but greater than wheat DDGS (Figure 1). It has been shown that protein fermentation influences gas production negatively [27], which can explain the lower gas production of DGGS as they contained 32.9% of CP. Values of AGPR of BS were similar to those for barley and sugar beet pulp, which are feeds rapidly degraded in the rumen, whereas AGPR values for white, Savoy, and red cabbage were similar to those measured for DDGS (Table 2). The DMED of all cabbages, estimated from PDMD and gas production parameters, was greater than that of the feeds used as reference, which was due to the high PDMD of cabbages (95.9, 89.3, 85.6, and 92.5% for BS, white, Savoy, and red cabbage, respectively) compared with that of reference feeds (89.8, 80.9, and 60.6% for barley, sugar beet pulp, and DDGS, respectively). These results indicated that the cabbages analyzed could be degraded extensively by ruminal microorganisms, even to a greater extent than feeds commonly used in ruminant feeding, namely barley, sugar beet pulp, and wheat DDGS.

The increased rates of gas production observed for BS would indicate that it was fermented more rapidly than the other cabbages, which is in agreement with the increased (*p* < 0.05) gas production and lower (*p* < 0.05) pH measured in the 24-h in vitro incubations (Table 2). However, there were no differences (*p* = 0.186) among cabbage samples in total VFA production, which was for all samples greater than that promoted by barley and DDGS, and only slightly lower than that measured for sugar beet pulp. The fermentation of BS produced lower (*p* < 0.05) acetate and greater (*p* < 0.05) propionate and butyrate proportions than the other Brassica vegetables, which resulted in lower (*p* < 0.05) acetate/propionate ratios for BS compared with white, Savoy, and red cabbages. Compared with the reference feeds, both VFA profile and acetate/propionate ratio in white, Savoy, and red cabbages were similar to those for sugar beet pulp, whereas the fermentation of BS was mainly characterized by the high propionate proportions.

The greater (*p* < 0.05) NH_3_-N concentrations in BS than in the rest of cabbages (Table 2) was in agreement with its greater CP content, and there was a positive relationship between CP content in the cabbages and NH_3_-N concentrations (*n* = 12; r = 0.914; *p* < 0.001). The greater (*p* < 0.05) proportions of minor VFA (calculated as the sum of isobutyrate, isovalerate, and valerate) for BS and Savoy cabbage compared with white and red cabbage also indicated a greater CP degradation, as minor VFA are produced in the deamination of branched amino acids [28]. Despite DDGS had greater CP content than the tested cabbages (Table 1), concentrations of NH_3_-N for wheat DDGS were lower (223 mg/L), which could be attributed to a lower degradability of wheat DDGS protein [28].

As shown in Table 3, values of 12-h in situ rumen degradability of DM were high **(**>86%) for all cabbages, with red cabbage samples having the lowest (*p* < 0.05) values. Rumen DM degradability values (*n* = 12) were positively correlated with sugars content (r = 0.745; *p* = 0.005) and negatively (*p* ≤ 0.011) with NDF, ADF, and lignin (r = 0.737, 0.785, and 0.700, respectively) content, reflecting the high degradability of sugars compared with fiber fractions. Rumen degradability of CP was greater than 91% for all samples, being close to 95% for white cabbage, which indicates that CP of the tested vegetables is rapidly and extensively degraded in the rumen. The high rumen CP degradability is in agreement with the high NH_3_-N concentrations observed for all samples in the 24 h in vitro incubations, and the positive correlation between CP content in the tested vegetables and NH_3_-N concentrations reported previously. A positive correlation was also observed when barley and sugar beet pulp were included in the data (*n* = 14; r = 0.970; *p* < 0.001), but the correlation was lower by including wheat DDGS values (*n* = 15; r = 0.742; *p* = 0.002), which was possibly due to the lower ruminal degradability of DDGS-CP compared with the rest of the samples, as previously reported [28].

There were marked differences among vegetables in the in vitro intestinal digestibility of both DM and CP, with BS having the greatest (*p* < 0.05) values and red cabbage the lowest (*p* < 0.05) ones. Intestinal digestibility values of both DM and CP were positively correlated (*p* ≤ 0.049) with CP and sugars content in the vegetables, and negatively (*p* ≤ 0.009) with the content of NDF, ADF, lignin, and neutral detergent insoluble CP, either expressed on DM basis or as a percentage of total CP. The low amount of by-pass protein in all cabbages (<10% of total CP; calculated as 100 minus the amount of degradable CP), together with the relatively low intestinal digestibility of this by-pass CP, indicate that the amino acids supply from cabbages CP to the host animal would be unimportant. Altogether, the results indicated that DM of all tested cabbages, but especially of BS, is a good source of N and rapidly fermented substrates for ruminal microbiota.

### 3.2. Experiment 2: In Vitro Fermentation and In Situ Degradability of Diets Containing Increasing Amount of Brussels Sprouts

Brussels sprouts were selected for Experiment 2 because they had the greatest DM, sugars, and protein content of all cabbages tested in this study. As shown in Table 4, BS replaced different amounts of corn, barley, and soybean meal with the objective that all diets had similar CP and NDF content. Potential gas production (A) increased linearly (*p* = 0.027), and AGPR tended (*p* = 0.053) to increase linearly with increasing BS amounts in the diet, but there were no differences (*p* ≥ 0.126) among diets in *c*, *Lag*, and DMED values (Table 5). The increased A values indicated that diets including BS were more extensively fermented than the control diet, which was in agreement with the increased VFA productions observed at both 8 and 24 h of in vitro fermentation. The trend to increased AGPR observed with increasing amounts of BS in the diet indicated that BS inclusion increased the rate of fermentation. This is in accordance with the more rapid production of VFA observed for BS16 and BS24 diets, as the proportion of total VFA at 24 h that was generated at 8 h of incubation was 52.5, 52.5, 56.2, and 58.2% for control, BS8, BS16, and BS24 diets, respectively. The inclusion of BS in the diet did not affect molar proportion of acetate (*p* ≥ 0.152), but at both incubation times, it increased linearly (*p* ≤ 0.001) the proportions of propionate and decreased linearly (*p* < 0.001) those of butyrate, resulting in lower (*p* < 0.05) acetate/propionate ratios for BS16 and BS24 compared with the control diet at 24 h of incubation. These results were consistent with the greater propionate proportions and lower butyrate (28.2 and 9.06 mol/100 mol, respectively) observed in the in vitro fermentation of BS compared with barley (22.2 and 17.5 mol/100 mol, respectively; Table 2).

Concentrations of NH_3_-N augmented linearly at 8 h (*p* = 0.033) as BS increased in the diet, and a trend (*p* = 0.090) was also detected after 24 h of incubation. The greater NH_3_-N concentrations in BS-containing diets were probably due to the faster degradation of BS protein compared with that of barley, corn, and soybean meal, as NH_3_-N is one of the main final products of protein degradation in the rumen. As shown in Table 6, in situ degradation of CP increased linearly (*p* = 0.008) from 0.167 to 0.242 h^−1^ with increasing proportions of BS in the diet. Besides, the soluble fraction of the CP (*a* fraction) of all diets, including BS, was greater (*p* < 0.05) than in the control diet, and this fraction is assumed to be immediately and completely degraded in the rumen in most protein evaluation systems [28,29,30,31]. Although there were no differences among diets in the CP potential degradability (*a* + *b*), the effective degradability calculated for a rumen digesta passage of 0.042 per h was greater (*p* < 0.05) for all diets containing BS than for the control diet (80.2, 83.1, 84.3, and 85% for control, BS8, BS16, and BS24, respectively). Finally, the linear increase (*p* = 0.048) observed in the proportions of minor VFA at 24 h of incubation as the proportion of BS augmented in the diet is also in agreement with previous results, as minor VFA are produced in the deamination of branched amino acids [28].

Brassica vegetables contain multiple secondary compounds, such as glucosinolates, phenolic compounds, carotenoids, and chlorophylls [14], but to our best knowledge, their possible effect on ruminal CH_4_ production has not yet been investigated. Including BS in the diet did not affect CH_4_ production at any time (*p* ≥ 0.453; Table 5), but a linear reduction (*p* = 0.039) of CH_4_/VFA ratio was observed at 24 h, and a trend (*p* = 0.092) was also observed at 8 h of incubation. These results were consistent with the greater propionate and lower butyrate proportions observed in the fermentation of the diets containing BS, as the production of propionate in the rumen is a hydrogen-consuming reaction, whereas production of butyrate generates hydrogen that is used by methanogenic archaea to produce CH_4_ [32].

The amount of in vitro AFOM increased linearly (*p* ≤ 0.001) with increasing BS amounts in the diet at both 8 and 24 h of incubation (Table 5), which is in agreement with the linear increase (*p* = 0.042) observed in DM effective degradability measured in situ (Table 6). In the in vitro technique, AFOM was greater in all BS-containing diets than the control one, while when incubated in situ, only BS24 diet had greater DM effective degradability than the control diet. Besides, in situ values were 1.15 times greater (values averaged across diets) than in vitro values, despite both values were calculated for a digesta retention time in the rumen of 24 h (in vitro incubations lasted for 24 h, and DM effective degradability was calculated for a rumen passage rate of 0.042% per h that corresponds to a retention time of 24 h). Differences between techniques can help to explain these results, as a substantial amount of undegraded samples (either soluble compounds or small particles) may be washed out of the nylon bags in the in situ technique zero-hour later, thus leading to an overestimation of effective degradability [33]. In contrast, the AFOM in the in vitro technique was estimated from VFA production, and all VFA produced were recovered at the end of the incubation, as there was no digesta passage in the in vitro system used in our study.

## 4. Conclusions

Discarded cabbages are high-moisture by-products, but their dry matter is rich in protein and sugars and is rapidly fermented in the rumen. Crude protein of all tested cabbages was rapidly and extensively degraded in the rumen (>91% after 12 h in situ incubation), indicating a negligible by-pass fraction, whose in vitro intestinal digestibility ranged from 61.4 to 90.2%. In vitro results indicated that dried Brussels sprouts could replace 24% of conventional feeds (barley, corn, and soybean meal) in the concentrate of a dairy sheep diet composed of 40:60 alfalfa hay:concentrate without producing negative effects on rumen fermentation. The inclusion of 24% of Brussel sprouts in the concentrate increased the effective degradability of diet DM and CP. Further research on the viability of the inclusion of these by-products in the diet in practice and to quantify animal response in both productive and economic terms is required, taking into account that cabbage wastes are high in sulfur and their feeding could increase the risk of sulfur toxicity and even modify the flavor of animal products.

## Figures and Tables

**Table 1:** Chemical composition of cabbage samples (*n* = 3; g/100 g dry matter unless otherwise stated) and of barley, sugar beet pulp, and wheat DDGS (dried distiller grains with solubles) used as reference (*n* = 1).

Item	Cabbages				SEM ^2^	*p*	Reference Feeds		
	Brussels Sprouts	White Cabbage	Savoy Cabbage	Red Cabbage			Barley	Sugar Beet Pulp	Wheat DDGS
Dry matter (%)	16.3 ^b^	5.64 ^a^	7.14 ^a^	6.60 ^a^	0.388	<0.001	89.9	89.1	92.2
Organic matter	92.2 ^b^	84.7 ^a^	86.4 ^a^	89.1 ^a^	0.44	<0.001	97.3	94.9	95.5
Crude protein (CP)	24.8 ^b^	19.5 ^a^	20.7 ^a^	19.8 ^a^	0.59	<0.001	12.4	9.44	32.9
Ether extract	2.90 ^a^	3.57 ^ab^	4.36 ^a^	4.54 ^a^	0.220	0.002	3.16	0.80	4.61
Sugars	41.4	35.3	34.2	27.2	3.35	0.095	3.69	13.5	6.97
Neutral detergent fiber (NDF)	17.5 ^a^	23.5 ^ab^	28 ^b^	25.4 ^b^	1.54	0.007	22.7	48	29.5
Acid detergent fiber	10.3 ^a^	15.4 ^ab^	17.3 ^b^	17.6 ^b^	0.751	<0.001	5.23	24.2	11.2
Lignin	0.25	1.53	1.97	2.43	0.574	0.119	1.22	2.16	3.33
Hemicellulose	7.24	8.15	10.7	8.87	0.845	0.087	19.6	23.9	18.3
Lignin (% of NDF)	1.43	6.20	7.01	9.40	1.914	0.092	5.37	4.49	11.2
NDICP ^1^ (% CP)	4.89	15.8	17.8	13.9	3.26	0.091	14.5	55	28.9

^a, b^ Within cabbages, means in the same row with different superscript differ (*p* < 0.05). ^1^ NDICP: neutral detergent insoluble crude protein expressed as g/100 g crude protein. ^2^ SEM: standard error of the mean.

**Table 2:** Gas production and fermentation parameters of cabbages (*n* = 3) and barley, sugar beet pulp, and wheat DDGS (dried distiller grains with solubles) used as reference (*n* = 1).

Item	Cabbages				SEM ^3^	*p*	Reference Feeds		
	Brussels Sprouts	White Cabbage	Savoy Cabbage	Red Cabbage			Barley	Sugar Beet Pulp	Wheat DDGS
Gas production parameters ^1^									
A (mL/g)	275 ^c^	219 ^a^	229 ^ab^	233 ^b^	3.27	<0.001	352	329	185
*c* (%/h)	5.55 ^c^	4.51 ^b^	4.75 ^b^	3.71 ^a^	0.17	<0.001	5	5.21	4.15
*Lag (h)*	2.59	2.44	3.03	2.73	0.187	0.169	2.85	3.83	0
AGPR (mL/h)	9.11 ^c^	6.17 ^ab^	6.49 ^b^	5.47 ^a^	0.239	<0.001	10.5	9.52	5.55
DMED (%)	58.4 ^b^	51.7 ^a^	49.1 ^a^	50.4 ^a^	0.69	<0.001	45	45.2	38.4
Fermentation parameters ^2^									
Gas (mL)	39.5 ^c^	34.2 ^a^	35 ^a^	36.8 ^b^	0.51	<0.001	49.9	43.9	26.3
pH	6.52 ^a^	6.61 ^bc^	6.65 ^c^	6.60 ^b^	0.011	<0.001	6.60	6.56	6.73
Total volatile fatty acids (VFA; µmol)	1632	1636	1612	1673	19.6	0.186	1452	1700	1311
Individual VFA (mol/100 mol)									
Acetate (Ac)	57.5 ^a^	62.6 ^b^	62.5 ^b^	62.9 ^b^	0.25	<0.001	56.6	65.5	53.4
Propionate (Pr)	28.2 ^b^	24.5 ^a^	24.3 ^a^	24.1 ^a^	0.25	<0.001	22.2	25.2	33.3
Butyrate	9.06 ^b^	8.18 ^a^	8.04 ^a^	8.31 ^a^	0.104	<0.001	17.5	6.93	6.34
Minor VFA	5.23 ^b^	4.73 ^a^	5.15 ^b^	4.65 ^a^	0.106	<0.001	3.70	2.31	6.96
Ac/Pr (mol/mol)	2.04 ^a^	2.55 ^b^	2.57 ^b^	2.60 ^b^	0.031	<0.001	2.55	2.60	1.61
NH_3_-N (mg/L)	288 ^c^	223 ^a^	257 ^b^	218 ^a^	5.4	<0.001	156	86.8	223

^a, b, c^ Within cabbages, means in the same row with different superscript differ (*p* < 0.05). ^1^ A: potential gas production; *c*: fractional rate of gas production; *Lag*: is the time needed to start gas production; AGPR: average gas production rate; DMED: dry matter effective degradability estimated for a rumen digesta passage of 0.042 per h; **^2^** Determined in 24-h incubations in batch cultures of ruminal microorganisms containing 200 mg of substrate dry matter; VFA: volatile fatty acids; Minor VFA: calculated as the sum of isobutyrate, isovalerate, and valerate; ^3^ SEM: standard error of the mean.

**Table 3:** In situ rumen degradability after 12 h of incubation and in vitro intestinal digestibility of dry matter (DM) and crude protein (CP) of cabbages (*n* = 3).

Item	Brussels Sprouts	White Cabbage	Savoy Cabbage	Red Cabbage	SEM ^1^	*p*
DM rumen (%)	93.1 ^b^	91.9 ^b^	89.5 ^ab^	86.9 ^a^	1.03	0.001
CP rumen (%)	91.6 ^a^	94.4 ^b^	92.7 ^ab^	91.8 ^a^	0.77	0.042
DM intestinal (%)	73.5 ^c^	45.7 ^b^	44.2 ^b^	35.3 ^a^	2.57	<0.001
CP intestinal (%)	90.2 ^c^	71.2 ^ab^	74.1 ^b^	61.4 ^a^	3.24	0.008

^a, b, c^ Means in the same row with different superscript differ (*p* < 0.05). ^1^ SEM: standard error of the mean.

**Table 4:** Ingredients and chemical composition of experimental diets containing increased amounts of Brussels sprouts (BS) used in Experiment 2.

Item	Diet			
	Control	BS8	BS16	BS24
Diet ingredients (g/100 g fresh matter)				
Alfalfa hay	40.0	40.0	40.0	40.0
Concentrate	60.0	60.0	60.0	60.0
Concentrate ingredients (g/100 g fresh matter)				
Brussel sprouts	-	8.0	16.0	24.0
Corn	32.0	28.0	25.0	23.0
Barley	30.0	28.8	26.5	23.0
Wheat	15.0	15.0	15.0	15.0
Soybean meal 46%	14.0	11.2	8.5	6.0
Wheat bran	7.0	7.0	7.0	7.0
Calcium soap	1.0	1.0	1.0	1.0
Calcium carbonate	0.5	0.5	0.5	0.5
Mineral/vitamin premix	0.5	0.5	0.5	0.5
Chemical composition of diets ^1^				
Dry matter	89.7	89.7	89.7	89.7
Organic matter	93.0	92.4	91.6	90.9
Crude protein	16.1	16.1	16.1	16.1
Neutral detergent fiber	31.5	31.6	31.7	31.6
Acid detergent fiber	15.9	16.1	16.4	16.7
Ether extract	4.1	4.1	4.0	4.0

^1^ Calculated from the analyzed composition of individual feed ingredients. All chemical fractions are expressed as g/100 g dry matter, excepting dry matter content which is expressed as g/100 g diet.

**Table 5:** Gas production and fermentation parameters of experimental diets containing 40% of alfalfa hay and 60% of concentrate with increased amounts of Brussels sprouts (BS; 8, 16, and 24% of concentrate).

Item	Diet				SEM ^2^	*p*	
	Control	BS8	BS16	BS24		Lineal	Quadratic
Gas production parameters ^1^							
A (mL/g DM)	280 ^a^	289 ^ab^	290 ^ab^	293 ^b^	3.47	0.027	0.388
*c* (%/h)	3.90	4.00	4.00	3.99	0.061	0.449	0.330
*Lag* (h)	1.10	0.87	0.62	0.64	0.21	0.126	0.574
AGPR (mL/h)	7.40	7.98	8.06	8.09	0.21	0.053	0.239
DMED (%)	40.6	41.9	42.2	41.9	0.62	0.147	0.214
Fermentation parameters (8 h)							
Total volatile fatty acids (VFA; µmol)	1284 ^a^	1391 ^ab^	1501 ^bc^	1561 ^c^	34.8	<0.001	0.512
Individual VFA (mol/ 100 mol)							
Acetate (Ac)	61.1	61.6	61.3	61.7	0.17	0.152	0.822
Propionate (Pr)	22.9 ^a^	22.9 ^a^	23.3 ^b^	23.6 ^b^	0.11	0.001	0.384
Butyrate	12.8 ^c^	12.5 ^bc^	12.3 ^b^	11.8 ^a^	0.12	<0.001	0.582
Minor VFA ^3^	3.11	3.01	3.04	2.95	0.061	0.141	0.929
Ac/Pr (mol/mol)	2.69 ^b^	2.71 ^b^	2.64 ^ab^	2.63 ^a^	0.022	0.019	0.454
NH_3_-N (mg/L)	143 ^a^	148 ^ab^	154 ^b^	155 ^b^	2.9	0.033	0.560
CH_4_ (mL)	6.90	7.24	7.02	7.36	0.330	0.453	0.991
CH_4_/VFA (mL/mmol)	5.40	5.22	4.68	4.71	0.311	0.092	0.739
AFOM (%) ^4^	31.3 ^a^	33.4 ^ab^	36.3 ^bc^	37.9 ^c^	0.84	<0.001	0.474
Fermentation parameters (24 h)							
pH	6.79 ^b^	6.75 ^ab^	6.74 ^a^	6.73 ^a^	0.01	0.004	0.394
Total VFA (µmol)	2446 ^a^	2650 ^b^	2673 ^b^	2684 ^b^	0.1	<0.001	0.008
Individual VFA (mol/ 100 mol)							
Acetate (Ac)	61.5	61.6	61.5	61.7	28.36	0.342	0.583
Propionate (Pr)	18.7 ^a^	19.0 ^ab^	19.3 ^b^	19.6 ^b^	0.12	<0.001	0.788
Butyrate	15.5 ^d^	14.9 ^b^	14.7^b^	14.1 ^a^	0.14	<0.001	0.931
Minor VFA ^3^	4.27 ^a^	4.53 ^ab^	4.53 ^ab^	4.57 ^b^	0.092	0.048	0.217
Ac/Pr (mol/mol)	3.31 ^c^	3.28 ^bc^	3.20 ^ab^	3.16 ^a^	0.0143	0.003	0.962
NH_3_-N (mg/L)	189	203	207	209	6.1	0.090	0.506
CH_4_ (mL)	14.9	15.1	14.8	15.2	0.41	0.804	0.846
CH_4_/VFA (mL/mmol)	6.10 ^b^	5.69 ^ab^	5.53 ^a^	5.65 ^a^	0.140	0.039	0.095
AFOM (%) ^4^	60.5 ^a^	64.0 ^b^	65.1 ^b^	65.4 ^b^	0.66	0.001	0.007

^a, b, c^ Means in the same row with different superscript differ (*p* < 0.05); ^1^ A: potential gas production; *c*: fractional rate of gas production; *Lag*: is the time needed to start gas production; AGPR: average gas production rate; DMED: dry matter effective degradability estimated for a rumen particulate outflow 0.042 per h; ^2^ SEM: standard error of the mean; **^3^** Minor VFA: calculated as the sum of isobutyrate, isovalerate, and valerate; ^4^ AFOM: fermented organic matter estimated from VFA production as proposed by Demeyer [25].

**Table 6:** In situ degradation parameters of dry matter and crude protein of experimental diets containing 40% of alfalfa hay and 60% of concentrate with increased amounts of Brussels sprouts (BS; 8, 16, and 24% of concentrate).

Item ^1^	Diet				SEM ^2^	*p*	
	Control	BS8	BS16	BS24		Lineal	Quadratic
Dry matter							
*a* (%)	33.6 ^a^	34.7 ^a^	37.9 ^b^	40.5 ^c^	0.33	<0.001	0.269
*b* (%)	45.6 ^b^	44.6 ^b^	42.5 ^a^	40.9 ^a^	0.34	0.001	0.628
*a* + *b* (%)	79.2	79.4	80.4	81.4	0.59	0.141	0.705
*c* (h^−1^)	0.262	0.240	0.206	0.238	0.0102	0.227	0.180
ED (%)	72.7 ^a^	72.7 ^a^	73.2 ^ab^	75.1 ^b^	0.67	0.042	0.192
Crude protein							
*a* (%)	35.7 ^a^	47.2 ^c^	45.8 ^b^	49.1 ^d^	0.23	<0.001	<0.001
*b* (%)	55.9 ^c^	44.5 ^b^	45.7 ^b^	42.2 ^a^	0.34	<0.001	0.001
*a* + *b* (%)	91.6	91.7	91.5	91.3	0.23	0.551	0.728
*c* (h^−1^)	0.167 ^a^	0.176 ^ab^	0.227 ^bc^	0.242 ^c^	0.0091	0.008	0.871
ED (%)	80.2 ^a^	83.1 ^b^	84.3 ^b^	85.0 ^b^	0.77	0.004	0.211

^a, b, c^ Means in the same row with different superscript differ (*p* < 0.05); ^1^ *a:* soluble fraction; *b:* non-soluble potentially degradable fraction; *c*: fractional degradation rate of *b* fraction; ED: effective degradability calculated for a rumen passage rate of 0.042 h^−1^; ^2^ SEM: standard error of the mean.
